# Identifying the Risk Factors of Allergic Rhinitis Based on Zhihu Comment Data Using a Topic-Enhanced Word-Embedding Model: Mixed Method Study and Cluster Analysis

**DOI:** 10.2196/48324

**Published:** 2024-02-22

**Authors:** Dongxiao Gu, Qin Wang, Yidong Chai, Xuejie Yang, Wang Zhao, Min Li, Oleg Zolotarev, Zhengfei Xu, Gongrang Zhang

**Affiliations:** 1 School of Management, Hefei University of Technology Hefei China; 2 Russian New University Moscow Russian Federation

**Keywords:** social media platforms, disease risk factor identification, chronic disease management, topic-enhanced word embedding, text mining

## Abstract

**Background:**

Allergic rhinitis (AR) is a chronic disease, and several risk factors predispose individuals to the condition in their daily lives, including exposure to allergens and inhalation irritants. Analyzing the potential risk factors that can trigger AR can provide reference material for individuals to use to reduce its occurrence in their daily lives. Nowadays, social media is a part of daily life, with an increasing number of people using at least 1 platform regularly. Social media enables users to share experiences among large groups of people who share the same interests and experience the same afflictions. Notably, these channels promote the ability to share health information.

**Objective:**

This study aims to construct an intelligent method (TopicS-ClusterREV) for identifying the risk factors of AR based on these social media comments. The main questions were as follows: How many comments contained AR risk factor information? How many categories can these risk factors be summarized into? How do these risk factors trigger AR?

**Methods:**

This study crawled all the data from May 2012 to May 2022 under the topic of *allergic rhinitis* on Zhihu, obtaining a total of 9628 posts and 33,747 comments. We improved the Skip-gram model to train topic-enhanced word vector representations (TopicS) and then vectorized annotated text items for training the risk factor classifier. Furthermore, cluster analysis enabled a closer look into the opinions expressed in the category, namely gaining insight into how risk factors trigger AR.

**Results:**

Our classifier identified more comments containing risk factors than the other classification models, with an accuracy rate of 96.1% and a recall rate of 96.3%. In general, we clustered texts containing risk factors into 28 categories, with season, region, and mites being the most common risk factors. We gained insight into the risk factors expressed in each category; for example, seasonal changes and increased temperature differences between day and night can disrupt the body’s immune system and lead to the development of allergies.

**Conclusions:**

Our approach can handle the amount of data and extract risk factors effectively. Moreover, the summary of risk factors can serve as a reference for individuals to reduce AR in their daily lives. The experimental data also provide a potential pathway that triggers AR. This finding can guide the development of management plans and interventions for AR.

## Introduction

### Background

Over the past few decades, the prevalence of chronic diseases has increased significantly, becoming a global public health concern. The World Health Organization has listed allergic diseases as one of the disease types that require priority research and prevention in the 21st century [1]. As a common chronic disease, allergic rhinitis (AR) is a multifactorial disease that is induced by environmental conditions or certain genes [2]. AR not only has a significant impact on individuals’ sleep, social life, and work attendance but also triggers comorbidities such as conjunctivitis, atopic dermatitis, and asthma [3]. Large-scale flow survey data showed that AR currently affects several people in China alone [4] and with an estimated prevalence between 15% and 20% worldwide [5]. The direct and indirect costs associated with the management of AR are also a significant burden on society. For instance, the total cost of AR in Sweden, with a population of 9.5 million, was estimated at €1.3 (US $1.41) billion annually [6]. These unexpectedly high costs could be related to the high prevalence of disease, in combination with the previously often underestimated indirect costs that arise from reduced work efficiency and absenteeism and the potential costs associated with treating AR comorbidities [6].

Currently, there is no cure for AR, and individuals need to avoid the disease risk factors such as exposure to allergens and inhalation irritants [7] during the long self-management process. Therefore, identifying AR risk factors can provide a reference for patients to help reduce the condition in their daily lives [8].

A plethora of studies have been proposed to identify AR risk factors. These studies recruited participants with symptoms of AR and control participants without AR symptoms from a specific age group or a particular geographical area. These studies collected demographic information, lifestyle habits, family history, comorbidities, and residential areas through questionnaires. Subsequently, they used correlation methods to explore the relationship between these data and AR, aiming to identify the risk factors for AR within the specified age group or geographical area [9]. However, these studies have 2 limitations. First, these studies specifically target certain age groups or geographical areas, and questionnaires can only gather data on specific pieces of information. Owing to the constraints of questionnaire surveys, it is challenging to identify potential risk factors that may be present in individuals’ daily lives. As a result, the risk factors identified through survey-based studies have a limited scope and are incomplete. As such, they provide limited insights for a broader patient population. Second, the survey-based approach demands a commitment to long-term investigation and a substantial effort to collect representative responses [10]. In contrast, collecting information from social media platforms can cover large geographical areas at a comparatively low cost [10]. Social media platforms allow users to share experiences and opinions on various topics [11,12], including personal health issues [13]. Over time, highly unstructured and implicit knowledge has been generated in communities where users frequently participate [14,15], which can provide daily health records that are difficult to obtain from traditional questionnaire surveys. Therefore, social media can become a potential source of information for identifying risk factors for diseases such as AR [16].

Text-mining techniques are an effective tool for using voluminous social media data [17]. Some studies have combined social media data analysis to obtain knowledge about disease risk factors [18,19]. However, the abovementioned studies on disease risk factors used only shallow text features such as the number of social media text items and word cooccurrences, which are not conducive to identifying disease risk factors in the context of colloquial and diverse user expressions [20]. In this study, we designed a text-processing framework to automatically identify risk factors from social media data [21]. We used social media comments to construct a natural language processing–based AR risk factor identification method, aiming to tackle the problems of omission and low accuracy in traditional disease-related information identification methods that rely solely on shallow text features such as word frequency.

To be more specific, we developed an AR risk factor identification method that integrates pretrained word embeddings with text convolutional neural networks (CNNs). The Word2vec algorithm has proven to be superior in text vector representation [20]. This is a prediction-based approach that predicts the neighboring words that are most likely to appear within a window size around a center word in a corpus, resulting in high-dimensional vector representations that capture semantic aggregation. As social media users may mention related topics, such as symptoms and treatments, when describing risk factors in their comments, we used a local context window to achieve better semantic aggregation of AR risk factors, a method that has been demonstrated to be effective for such aggregation. In addition, using the Skip-gram model to train word pairs enables the incorporation of word thematic information, thus improving attention to risk factor phrases. The convolutional network can convolve the text in the word vector dimension and extract critical information through the max-pooling layer operation. In addition, this study used a clustering method with review mechanisms to concentrate on a large amount of text that contains risk factors within the observable range, thereby ensuring the usefulness of the content obtained through text mining.

Our main contributions were as follows:

1. First, this study proposed a framework (TopicS-ClusterREV) based on natural language processing for identifying the risk factors of AR. We used pretrained word embeddings and text convolutional networks to process social media text. Our model can identify more risk factors from social media comments with high accuracy and recall. To the best of our knowledge, this is the first study to use natural language processing techniques to identify risk factors for AR in social media comments.
2. Second, this study proposes a topic-enhanced word-embedding model. TopicS enhances the thematic information of words by adding a task that predicts the theme to which the center word belongs. This generates high-dimensional word vector representations with semantic aggregation and theme enhancement. We trained 2 types of word vectors using both the Skip-gram and TopicS models and separately input them into each risk factor classifier. The results showed that TopicS outperformed the baseline on the text classification task, demonstrating the effectiveness of our topic-enhanced word-embedding model.
3. Finally, we introduced automatic and manual review mechanisms to improve the single-pass algorithm, which allowed us to effectively identify and focus on a large amount of text that contains risk factors within the observable range. We ultimately identified 28 categories of risk factors including the common risk factors that lead to most individuals developing symptoms and previously overlooked risk factors that were not within the scope of previous research.

### Identification of AR Risk Factors Through Surveys

AR has become a major global issue with a substantial increase in its prevalence in recent years. In Europe, the prevalence of AR among Danish adults progressively increased from 19% to 32% over the past 3 decades [22]. Understanding the risk factors, such as genetic, environmental, and lifestyle factors, helps in the management of AR, thus motivating many studies to focus on identifying potential risk factors. These studies are summarized in Table 1. From Table 1, we observed that the previous studies were based on survey methods, including cross-sectional surveys, cohort studies, and case-control studies.

**Table 1:** Summary of the literature related to risk factors for allergic rhinitis (AR)^a^.

Study, year	Method	Risk factors
Chiang et al [23], 2016	Case control	Exposure to sulfur dioxide
Kurganskiy et al [24], 2021	Cross-sectional	Grass and tree pollen
Lee et al [25], 2021	Cross-sectional	The widespread use of industrial chemicals
Paciência et al [26], 2020	Cross-sectional	Indoor decoration materials containing volatile organic chemicals
Saulyte et al [27], 2014	Case control	Active smoking
Kong et al [28], 2021	Cohort	Stress
Han et al [29], 2016	Cross-sectional	Obesity
Kanazawa et al [30], 2018	Cross-sectional	TYRO3 gene
Alm et al [31], 2014	Cross-sectional	Using antibiotics in the first week after birth

^a^We searched for the literature related to AR risk factors and presented 9 papers from the past decade to showcase the methods and the identified risk factors.

These studies typically recruited participants with symptoms of AR and control participants without AR symptoms from a specific age group or a particular geographical area, collected demographic information through questionnaires, and then conducted correlation analysis, such as logistic regression, to explore the relationship between those metadata and AR [32]. For instance, Gao et al [9] conducted a cross-sectional survey to investigate the prevalence and risk factors of adult self-reported AR in the plain lands and hilly areas of Shenmu City in China and analyzed the differences between regions. The content of the web-based questionnaire included demographic factors, smoking status, the comorbidities of other allergic disorders, family history of allergies, and place of residence. The unconditional logistic regression analysis was used to screen for factors influencing AR. Finally, they found that the prevalence of AR existed in regional differences. Genetic and environmental factors were the important risk factors associated with AR. However, these studies have 2 limitations. First, these studies specifically targeted certain age groups or geographical areas, and questionnaires can only gather data on specific pieces of information. Owing to the constraints of questionnaire surveys, it is challenging to identify potential risk factors that may be present in individuals’ daily lives. As a result, the risk factors identified through survey-based studies have limited scope and are incomplete and they may provide limited insights for a broader patient population. Second, the survey-based approach demands a commitment to long-term investigation and a massive effort to collect representative responses [10].

### Identification of Disease Risk Factors From Social Media Through Text Mining

Social media sites provide a convenient way for users to continuously update their day-to-day activities, which allows large groups of people to create and share information, opinions, and experiences about health conditions through web-based discussion [11]. Hence, social media can be considered a new data source to assess population health. As shown in Table 2, some studies have combined text-mining techniques to classify and summarize voluminous social media data to obtain knowledge about chronic disease risk factors. Zhang and Ram [33] extracted behavioral features from Twitter posts of asthma users using keywords from an existing knowledge base. Griffis et al [34] collected 25,000 tweets containing and not containing diabetes, identified 5000 common words, used logistic regression to determine which common words were high-frequency expressions of diabetes, and finally grouped these high-frequency words using latent Dirichlet allocation to obtain the risk factors for diabetes. Schäfer et al [35] used syntactic analysis to identify portions of risk factors occurring before or after causal terms, grouped these portions using latent Dirichlet allocation, and obtained the risk factors for gastric discomfort. Pradeepa et al [19] performed clustering on stroke-related tweets using the Probability Neural Network, used the Apriori algorithm to identify frequent word sets related to risk, and thus identified risk factors for stroke [19]. In addition to the aforementioned approaches that use shallow text features such as keywords, frequent word sets, high-frequency words, and syntactic features for disease risk factor identification, other studies [36-38] trained risk factor classifiers using machine learning methods such as Naive Bayes, Maximum Entropy Model, and Naive Bayes Classifier–Term Frequency Inverse Document Frequency. These classifiers predict the presence of risk factors in text based on discrete vector representations such as bag-of-words and n-gram.

**Table 2:** Summary of the literature related to disease risk factors based on social media data^a^.

Study, year	Social media platforms	Data	Methods	Features	Diseases	Identified risk factors
Zhang and Ram [33], 2020	Twitter	Posts	Semisupervised learning	Knowledge base	Asthma	Behavioral attributes
Griffis et al [34], 2020	Twitter	Posts	LDA^b^	Word frequency	Diabetes	BMI, waist, drugs, alcohol, and obesity
Schäfer et al [35], 2020	Doctissimo, Aufeminin	Answers	LDA	Syntactic	Gastrointestinal discomfort	Food and psychological factors
Pradeepa et al [19], 2020	Twitter	Posts	A priori	Frequent word set	Stroke	Lifestyle, family history, heart disease
Alswedani et al [39]	Twitter	Posts	Keywords list	Word frequency	Psychological health	Social and economic factors, individual factors, diseases and disorders
Chung et al [40]	Telegram (app)	Time	MLP^c^	Meta data	Respiratory diseases	Pollution
Neisani Samani et al [41]	Twitter	Posts	Content analysis methods	Word frequency	Oropharyngeal cancer	Drinking, smoking

^a^We searched for studies related to identifying disease risk factors based on social media data. We found 7 papers from the past decade, highlighting the social media platforms, data, methods, features, diseases, and risk factors involved in research.

^b^LDA: latent Dirichlet allocation.

^c^MLP: multilayer perceptron.

The current methods for identifying disease risk factors on social media fall into 2 categories: shallow text feature methods and discrete word vector representations. Shallow text feature techniques often fail to capture important risk factors resulting in low accuracy, whereas discrete word vector approaches struggle to keep up with the dynamic vocabulary of social media text, missing new words, and trending expressions, thus inadequately representing the information conveyed.

### Word Embedding and Text Classification Based on Deep Learning

Natural language processing technology promotes text analysis based on social media comments [39]; this technology can learn the deeper semantic features of the comment text and the features that are consistent with the current context, according to different training corpus, to input a better text vector representation for downstream classification tasks. Some researchers have used large-scale pretrained language models [40], global matrix decomposition [41], and local context windows [42] for text vector representation. Local context windows are more suitable for semantically aggregating AR risk factors [43]. Skip-gram and Continuous Bag-of-Words Model (CBOW) are prediction-based methods that learn the semantic representation of a center word by predicting the most likely neighboring words within a window size in a corpus. When users narrate risk factors in their comments, they may also mention symptoms, treatments, and other topics. These global contexts may dilute the key features of the risk factors expression. CBOW averages the context words to predict the target word and tends to predict high-frequency words in the corpus. In contrast, Skip-gram gives each word a chance to be a center word, making it better at predicting rare words compared with CBOW [44]. Therefore, in situations where social media users express a wide variety of ideas, the Skip-gram model can yield satisfactory outcomes. Moreover, the Skip-gram approach uses word pair training, which facilitates the incorporation of topic information into words [45], resulting in the generation of high-dimensional word vectors that feature semantic aggregation and topic enhancement. Therefore, we selected Skip-gram as the word-embedding model for our study.

Text classification has evolved to deep learning models, mainly including CNN-based models [46], recurrent neural network (RNN)–based models [47], and transformer models [48]. For the CNN algorithm, convolutional networks can convolve text on the word vector dimensions and extract key information through pooling layer operations. Consequently, this algorithm is capable of using essential data for classification tasks. Therefore, we used TextCNN for classifier training and evaluated the performance of RNN and transformer models on this task.

## Methods

### Framework

**Figure 1:**
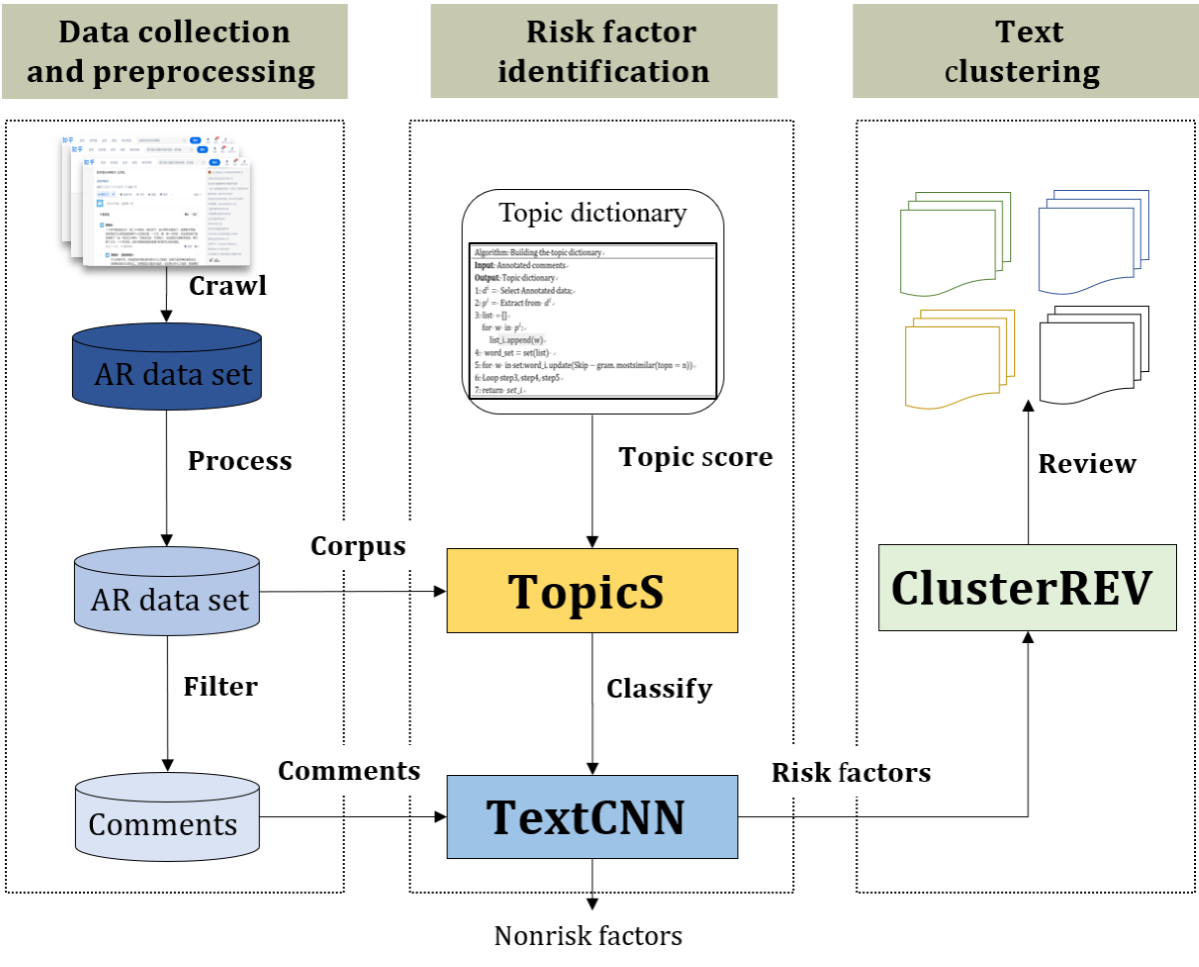
Allergic rhinitis (AR) risk factor identification method based on the topic-enhanced word-embedding model (TopicS-ClusterREV). The figure shows the research framework of our study. The framework consists of 3 parts. The first part is data collection and processing aimed at obtaining a clean data set. The second part is risk factor identification, which includes the proposed TopicS method and training of a risk factor classifier. The third part is text clustering and keyword extraction, which uses the ClusterREV method to cluster identified risk factors and extract keywords from every category.

The framework used in this study consisted of 3 parts as shown in Figure 1. The first part was data collection and processing, aimed at obtaining a clean data set. The second part was risk factor identification, which included the proposed TopicS method and training of a risk factor classifier. The implementation steps were as follows: (1) semiautomatically constructing a risk factor topic dictionary, (2) generating high-dimensional word vectors enhanced by TopicS-generated topics, and (3) vectorizing annotated text and training a risk factor classifier. The third part is text clustering and keyword extraction, which uses the ClusterREV method to cluster the identified risk factors and extract keywords from every category.

### Data Set

Zhihu is a Chinese social media platform where people discuss topics in an web-based forum format. In May 2022, the Zhihu subcommunity *allergic rhinitis* had 1.04 million discussions. The posts on this social media platform allow other users to comment [49], and people can explain their situations to provide support or seek help effectively. Therefore, these comments provide a rich source of data for investigating the risk factors reported by different users [50]. In this study, we trained domain-specific word representations based on experimental data. A relatively domain-specific input corpus [51] is better at extracting meaningful semantic relations than a generic pretrained language model [52]. We crawled all the data from May 2012 to May 2022 under the topic *allergic rhinitis* on Zhihu, obtaining a total of 9628 posts and 33,747 comments, including the post ID, comment ID, and post and comment content.

In this study, we preprocessed the data through regularization, stop word removal, and word separation. First, we removed special symbols, such as URLs and emoticons, in the comments through regularization and stop word removal to reduce the interference of noise with the text analysis task. Then, we compiled a dictionary of 169 specialized terms, including types of AR, medications, and comorbidities, to reduce the probability of incorrect word segmentation. After word separation, we obtained a lexicon of 68,863 words and ranked the words according to the number of occurrences. We found that the top 10,000 words accounted for 94.83% of the total words, suggesting that many words recurred and a relatively simple word vector could effectively train the model [53]. This further confirms the efficacy of our decision to use Skip-gram as the foundational model.

We observed ultrashort comment noise in the comments (eg, “Thank you!”). It is important to note that these ultrashort comments do not include any personal medical information. The ultrashort comments were filtered, resulting in 33,039 valid comments. This operation can effectively minimize the impact of noise on downstream text classification tasks. Table S1 in Multimedia Appendix 1 presents the examples of valid comments.

### Annotation

**Figure 2:**
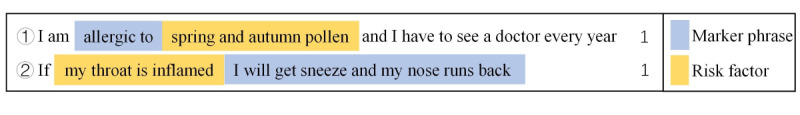
Examples of short text annotation. The figure shows examples of data labeled as 1 including the text and label. In the figure, phrases with a blue background indicate those that were specifically noted during manual annotation, and the presence of these marker phrases often suggests potential risk factors in the sentence. The yellow background highlights the risk factors in the text.

The data must be labeled before supervised learning and then trained end to end. If a comment directly mentions an allergen or indicates a condition that leads to the appearance or worsening of symptoms, the comment will be labeled as 1, indicating the presence of risk factors, as shown in Figure 2.

We randomly chose 2030 comments from the 33,039 comments, and 3 researchers labeled each comment as containing or not containing risk factors. To ensure high interannotator consistency, all 3 researchers annotated all 2030 comments. In cases with uncertainty in labeling, the 3 researchers discussed and arrived at a final label. After annotating and eliminating comments with religiously controversial content, 2000 labeled comments remained, consisting of 996 comments containing risk factors and 1004 comments not containing risk factors. The data set was divided into a 90% training set and a 10% test set. The 90% training set was further divided into 10 subsets, with 9 subsets used for training and the remaining subset used for validation, performing 10-fold cross-validation.

### Topic Dictionary Construction

We used a combination of manual labeling and similarity calculation to identify keywords related to risk factors. Subsequently, we constructed a table of topic words using a semiautomated approach. The process of constructing the dictionary is depicted in Textbox 1 and is as follows: (1) label 400 randomly selected comments as described in the *Annotation* section, thereby obtaining 198 comments with risk factors; (2) extract risk factor phrases from annotated comments; (3) obtain risk factors topic word list; (4) remove duplicate word list, and the words in the current topic are used as seed words, *word_set*; (5) use Skip-gram to find the top similar words to expand the topic words; (6) repeat steps 3 through 5 to expand the topic word; and (7) finally, obtain the topic words for the risk factor. A large weight was assigned to the risk factor theme words. Table S2 in Multimedia Appendix 1 shows examples of the risk factor topic dictionary.

**The algorithm for building the topic dictionary. This textbox outlines the algorithm process for building the topic dictionary with explanations for each step provided in the text.**

Input: annotated comments

Output: topic dictionary

1. d^i^= Select Annotated data;

2. p^i^= Extract from d^i^

3. list =

for w in p^i^:

list_i.append(w)

4. word_set=set(list)

5. for w in set: word_i.update(Skip-gram.mostsimilar(topn=n))

6. Loop step3, step4, step5

### Ethical Considerations

As the use of text data from social media involves user privacy, this study adopted the following steps for deidentification: (1) We removed user account information and retained only anonymous comment information. (2) We used regular expressions to match and delete URLs and email addresses in the comments. (3) During the annotation process, annotators received only text that did not involve personal information. To evaluate the quality of deidentification, we randomly selected 500 text items for manual inspection and did not find any instances containing personal identity information. Our data are sourced from public discussions on Zhihu, a social media platform that can be accessed without registration. We followed strict ethical research protocols similar to the guidelines by Eysenbach and Till [54]. In addition, to protect the anonymity of participants, we have implemented measures including the removal of user information and avoiding verbatim quotations to prevent identification through search engines, protecting the privacy and security of personal data. It should be mentioned that our study was focused on the post level; we do not anticipate any negative ethical impact from our analysis.

### Topic-Enhanced Word Embedding

**Figure 3:**
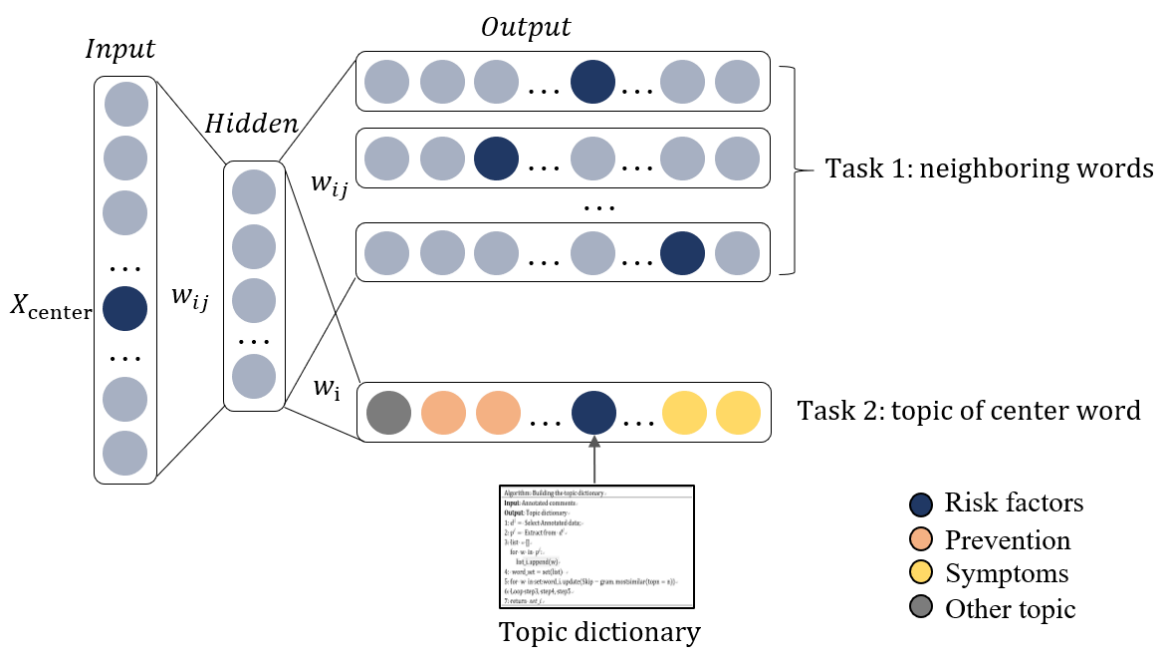
Topic-enhanced word-embedding model (TopicS). The figure illustrates the vector changes within the TopicS model. The rectangular boxes in both the input and output represent one-hot vectors. Within the input, the dark blue circles signify the center words, representing a value of 1, whereas the light blue circles denote other words in the training text, with a value of 0. For the output’s task 1, the dark blue circles depict context words surrounding the center word, signifying a value of 1, whereas the light blue circles represent noncontext words with a value of 0. The various colored circles in the output’s task 2 indicate the topics to which the center word belongs. If it pertains to the risk factor topic, it is marked by a dark blue circle, symbolizing a value of 1, whereas circles of other colors represent a value of 0.

TopicS performed 2 tasks during training, as shown in Figure 3. The first task was to predict the neighboring words within the window of the central word. The second task was to predict the topic of the central word; the topic dictionary used for this purpose is described in the *Topic Dictionary Construction* section.

The specific formula calculations for the loss function design, parameter updates, and error backpropagation of TopicS are explained subsequently.

First, we defined the loss function. For each word in the corpus, we used it as the central word for a sliding operation with a window size of *c*; let *S* be the training sequence (*w_1_,w_2_,...,w_T_*), whereas *w_i_* denotes the *i*th word in the sequence. The subscript *T* represents the total number of unique words in the corpus. In addition to predicting the contextual word of the central word, we must also predict the topic score of the central word. Therefore, the loss function comprised 2 parts: *L_cont_* and *L_topic_*, and the overall loss was denoted by *L_s_*. Our training objective was to minimize the loss function:



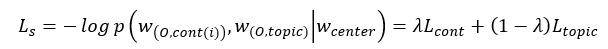



The initial word vector was represented as one-hot vector. The central word was denoted as *w_center_*, the surrounding words of the central word were denoted as *w_(O,cont(i))_*, and the central word topic information was denoted as *w_(O,topic)_*. The weight parameter *λ* was used to balance the loss of *L_cont_* and *L_topic_*. After sliding the window to browse the entire corpus to average all loss window losses, error backpropagation was performed to update the parameters. The input matrix was represented as the central word vector. The actual loss function can be expressed as 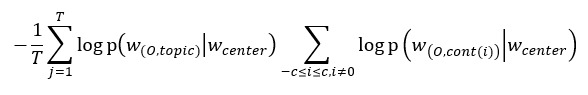


Second, we introduced the rules for updating parameters. The parameters were updated through error backpropagation 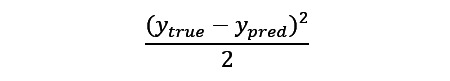 Here, *y_true_* represents the true value of the contextual words in the window, and *y_pred_* represents the predicted value.

Finally, we can update the word representation.

### Text Classification

**Figure 4:**
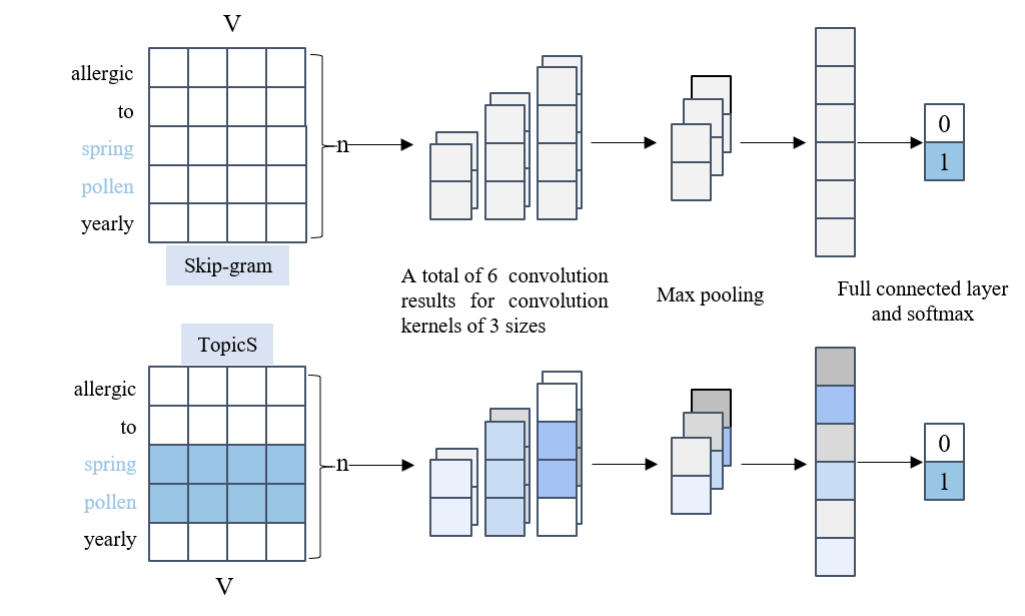
Framework of the classification model with different word embedding. This figure illustrates the TextCNN modeling process for text vectorization using both the skip-gram and TopicS techniques. In the example sentence, “spring” and “pollen” are highlighted as risk factors. These words are represented by blue squares in TopicS, suggesting that TopicS incorporates topic information, unlike the skip-gram method. These thematic data are subsequently integrated into the convolution, max-pooling, and softmax procedures to enhance the model’s classification capabilities.

In this study, we chose TextCNN as the classification model. In the risk factor identification task, some key semantic information is more important, and TextCNN can efficiently use the key information for classification with minimal cost consumption. We represented the manually annotated text as a vector matrix using high-dimensional word vector representations trained by the TopicS model, which aggregates local contextual and topic information and uses it as input for the TextCNN model. Then, the TextCNN algorithm leverages convolutional kernels of different sizes to extract multiple n-gram text features and uses convolutional operations in a fixed window to combine word representations to capture local information. Our input word vector combined the topic information of words, and the most important features in the convolution operation can be extracted using the maximum pooling operation as shown in Figure 4.

### Clustering With a Review Mechanism

The clustering task is to group similar risk factors. In this study, a large amount of text containing risk factors was clustered into a manually observable number of categories, making it easier to comprehend their content. This study enhances the single-pass algorithm and integrates it with a manual review to cluster the risk factors identified in the text classification, ensuring the validity of the clustering results. The main concept of single-pass clustering [55] is to match informational text items based on their similarity values without the need to determine the number of clusters in advance. This makes it suitable for clustering tasks with an unknown number of clusters. However, traditional single-pass clustering uses only one-loop traversal, which may result in previously entered text items completing the traversal earlier. This can cause their similarity to the previous topics to be slightly lower than the threshold and lead to them being recreated as new categories, ultimately affecting the clustering effect.

**Figure 5:**
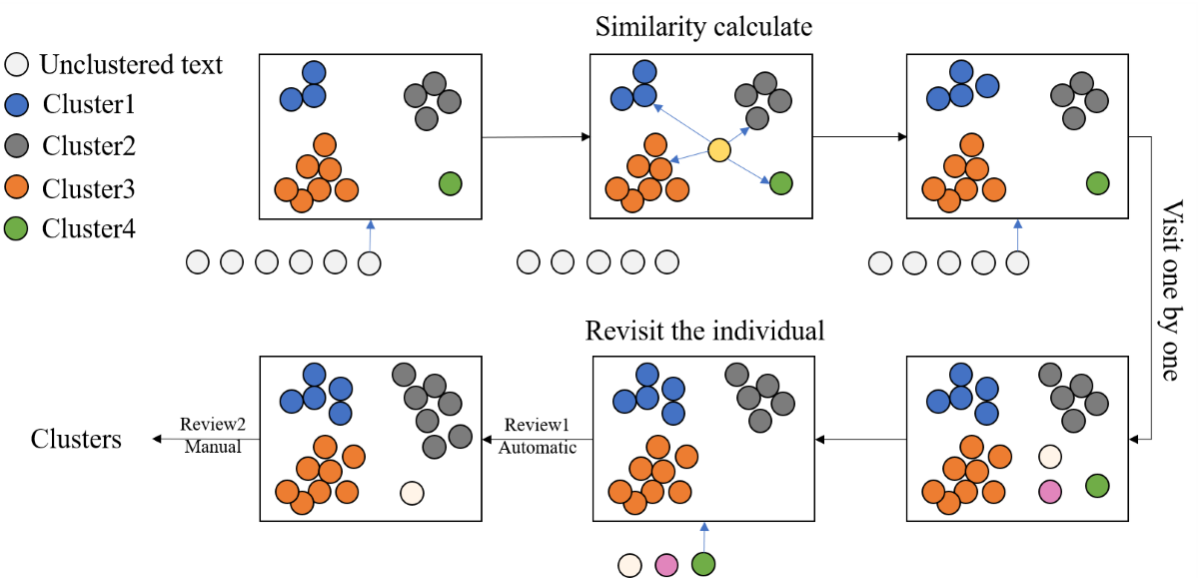
Cluster method with review mechanisms (ClusterREV). This figure depicts the process of the ClusterREV algorithm. The rectangular boxes represent category state transitions. The circles below the rectangles indicate the texts awaiting clustering. The algorithm assesses the distance between the current text and existing categories, classifying the text based on the minimal distance and a set threshold. Once all texts have been clustered, texts within a solitary category undergo automatic review. Finally, we manually reviewed the clustering results.

As shown in Figure 5, we improved the single-pass algorithm by retraversing the categories that were clustered separately after all the text items had been traversed to handle any missed text. After the automated clustering was completed, we conducted a manual review to ensure the reliability of the clustering.

Moreover, this study uses a keyword cloud visualization of category content to quickly understand the themes and characteristics of each cluster and compare the differences between different clusters. TextRank [56] was selected to extract category keywords, which considers only the voting scores of words in a single document; common words that frequently appear in a single document easily obtain high scores [57]. We treated each category as a single document for keyword extraction. As risk factors appear more frequently in categories, TextRank can effectively extract risk factors and surrounding words, preserving category content information as much as possible and reflecting the true content of the risk factors.

## Results

### Overview

In this section, we present the performance of the classifier and the findings based on the categorization of all the comments in the clean data set using the classifier. Our approach involved visualizing the clustering results of the risk factors to comprehend the primary elements of these factors. We also explored the pathogenic mechanisms associated with these risk factors.

### Classifier Performance

We used standard text-mining evaluation metrics such as accuracy, precision, recall, and *F*_1_-score to evaluate the performance. Precision assesses how many risk factors the model identifies correctly, and recall measures how many risk factors the model can identify on its test set. As we aimed to identify as many AR risk factors as possible to provide comprehensive references for individuals, recall was more important than precision in our study.

We set 7-word embedding dimensions ranging from 100 to 400. Table 3 displays the classification results of the TextCNN classification model with the 7 dimensions of Skip-gram and TopicS word vectors. In addition, TextRNN and transformer models were evaluated with the 7-word embedding dimensions of TopicS or Skip-gram, as shown in Tables S3 and S4 in Multimedia Appendix 1; the classification models performed better when the word-embedding dimension was 100 or 150, as shown in Table 4, which includes the results with best-performing dimensions. This study conducted word representation learning on a domain-specific input corpus, where low dimensionality was found to be sufficient to represent the features of the corpus [58]. Moreover, TopicS not only improved precision but also significantly increased recall for all 3 models, as shown in Table 4.

**Table 3:** Word-embedding dimension parameters with TextCNN.

Evaluation metrics and model			100		150		200	250			300		350		400
Accuracy (%)															
	Skip-gram	93.75		94.85		94.00			93.15	93.80		93.49		93.40	
	TopicS^a^	95.10^b^		96.10		94.80			94.45	94.95		95.25		95.40	
Precision (%)															
	Skip-gram	91.92		95.32		94.07			93.16	93.33		93.28		93.09	
	TopicS	96.32		95.95		95.56			94.11	95.20		95.87		96.53	
Recall (%)															
	Skip-gram	94.00		94.50		94.00			93.30	94.40		93.90		93.90	
	TopicS	95.90		96.30		94.00			94.90	94.70		94.60		94.20	
*F*_1_-score (%)															
	Skip-gram	93.90		94.88		93.95			93.17	93.84		93.48		93.44	
	TopicS	95.09		96.10		94.77			94.48	94.94		95.22		95.34	

^a^TopicS represents the topic-enhanced word-embedding model proposed in this paper.

^b^Italicization represents that the metrics of TopicS are better than Skip-gram for each metric.

**Table 4:** Accuracy, precision, recall, and *F*_1_-score of Skip-gram and TopicS with different classification models.

Model (Embed_size^a^)			Accuracy (%)		Precision (%)		Recall (%)	*F*_1_-score (%)		Time (s)
TextCNN										
	Skip-gram (150 dimens)	94.85		95.32		94.50		94.88	40.30	
	TopicS (150 dimens)	96.10^b^		95.94		96.30		96.10	35.23	
TextRNN										
	Skip-gram (150 dimens)	94.85		95.32		94.50		94.88	40.30	
	TopicS (150 dimens)	96.10^b^		95.94		96.30		96.10	35.23	
Transformer										
	Skip-gram (100 dimens)	85.45		85.06		78.80		81.13	55.16	
	TopicS (150 dimens)	90.70		90.90		90.60		90.68	49.32	

^a^Embed_size represents the word-embedding size.

^b^Italicization represents that the metrics of TopicS are better than Skip-gram for each model.

Table 4 shows that TextCNN has the highest accuracy and recall rate among the 3 classification models. The highest accuracy achieved by our classification model was 0.9594, which used a 150-dimension word-embedding representation obtained from TopicS. In other words, TextCNN can detect more risk factors and minimize the loss of risk factors resulting from classification errors. The CNN model can extract key information similar to n-grams in sentences. The combination of TopicS and TextCNN can enhance topic information and achieve an aggregation effect. Our implementation process was the simplest and consumed the least resources. Our model examined 30,372 comments and identified 5221 comments containing risk factors.

### Risk Factor Clustering Results

We clustered the text items obtained from the text classification into 28 categories and extracted keywords from each category to better understand the content. Table 5 shows the top 5 categories and their corresponding keywords. The complete list can be found in Table S5 in Multimedia Appendix 1. We used category 1 as an example to explain the category formation process and demonstrate the validity of the qualitative results. As shown in Table 4, we labeled category 1 as *Season* based on the analysis of keyword weights and relative comments. The comments related to this category focused on seasonally induced AR, with factors such as changes in the weather during seasonal transitions and colder temperatures during winter, which can exacerbate symptoms. We also counted the number of text items in each category and found that seasonal, regional, mites, and weather changes were common risk factors for most patients. In addition, patients’ unhealthy lifestyle habits were also important risk factors widely present in research investigations. Furthermore, most patients reported experiencing symptoms at specific times (eg, “morning”), but researchers have paid little attention to the timing of symptom occurrence (which we refer to as time points).

**Table 5:** Category keyword distribution and visualization.

Category	Top 10 words (weight)	Word cloud	Number of text items
Season	Summer (0.031), winter (0.031), season (0.02), season change (0.014), spring (0.013), autumn (0.013), seasonal (0.013), nose (0.011), air conditioning (0.01), month (0.007)	Multimedia Appendix 2	852
Region	Beijing (0.035), Shenzhen (0.019), air (0.010), Wuhan (0.010), Guangdong (0.01), city (0.009), Shanghai (0.009), dust mites (0.009), nose (0.009), university (0.009)	Multimedia Appendix 3	644
Mites	Dust mites (0.111), mites (0.045), dust (0.02), allergy (0.012), pollen (0.008), allergens (0.007), effect (0.006), child (0.005), nose (0.005), cold air (0.005)	Multimedia Appendix 4	608
Weather	Cold air (0.071), weather (0.023), temperature (0.021), nose (0.02), winter (0.009), changes (0.008), air (0.008), alternate (0.008), summer (0.007), air conditioning (0.007)	Multimedia Appendix 5	538
Other diseases	Cold and flu (0.095), conjunctivitis (0.019), urticaria (0.016), nose (0.01), asthma (0.009), cough (0.008), eczema (0.008), winter (0.004), eyes (0.004), physique (0.004)	Multimedia Appendix 6	372

### The Possible Pathway of Several Risk Factors Triggers AR

We referred to the relevant literature on the risk factors associated with AR to confirm whether the extracted risk factors were consistent with the general medical consensus. Our findings are novel compared with those in the literature [59]. Previous survey-based studies have explored only the correlation between risk factors and AR, whereas our experimental data provide insight into the potential pathogenesis of reported risk factors. The following section provides a theoretical discussion of potential pathways for several risk factors that trigger AR:

- *Season*: (1) seasonal risk factors are manifested in pollen allergens. Tree allergens such as elm and cypress pollen are prevalent in early spring, followed by ash, pine, and birch pollen in late spring. In summer, grasses, artemisia, and flowering plants grow vigorously owing to increased rainfall, leading to increased pollen spread from these plants. In autumn, weeds account for the largest proportion of pollen allergens. (2) Different climatic conditions in different seasons contribute to the development of allergies. For example, in early spring, frequent cold and high-pressure air activity in East Asia causes intense atmospheric circulation, resulting in alternating hot and cold temperatures that impair the immune regulatory function of the human body, leading to increased allergy attacks. In autumn, changeable weather, large temperature differences, and sunlight and UV radiation can stimulate allergic reactions in people with weak lungs or those who are prone to AR. In addition, seasonal changes and increasing temperature differences between day and night can disrupt the human immune system.
- *Poor habits*: major keywords for this topic were “smoking,” “staying up late,” and “resistance.” (1) Habits such as staying up late, lack of exercise, smoking, and alcohol abuse can weaken immunity and resistance. Gangl et al [60] found that smoking can reduce the integrity and barrier function of respiratory epithelial cells, thereby making smokers more susceptible to allergens. (2) An irregular diet can damage the spleen and stomach, which is also a key factor in the development of AR. (3) The frequent use of air conditioning in summer can cause nasal mucosa irritation owing to temperature fluctuations. Long-term exposure to adverse stimuli can cause dryness of the nasal cavity and weaken the resistance of the mucosal epithelium, which may lead to AR.
- *Allergens*: we grouped clusters that included mites, plants, food, animals, and mold as allergens. (1) The findings of this study suggest that dust mites are the primary allergen, and exposure to a certain concentration of indoor dust mites can lead to AR. The ideal humidity level for dust mite growth is between 75% and 80%, and dust mites tend to thrive during spring and autumn and in warm and humid environments. Studies have shown that a large number of dust mites may be attached to uncleaned air conditioning filters, confirming that air conditioning is an important route of transmission for household dust mites [61]. (2) Allergenic pollen species are closely related to regions and seasons, and some regions now provide pollen concentration and allergy index broadcasts based on meteorological conditions, which is highly convenient for individuals experiencing allergy. (3) Food allergens such as milk, eggs, wheat, soybeans, and peanuts can also trigger AR. (4) Apart from dust mites, other perennial indoor allergens include animal dander, cockroach excrement, and molds.
- *Outdoor environment*: this topic had “dust,” “air quality,” “trust,” and “allergen” as high scoring words. (1) Various substances present in the outdoor environment can trigger AR. Industrialization has increased the content of aromatic hydrocarbon particles, ethanol, and formaldehyde in diesel exhaust, which can damage the mucous membrane and serve as a strong stimulus for AR attacks. (2) Air pollution can affect the distribution of allergens such as mold and pollen. In hazy weather, allergens tend to stay in the air longer, increasing the chance and duration of contact with the human body and leading to AR. (3) High winds can raise dust, pollen, mites, bacteria, and other allergenic factors, increasing their concentration in the air and making it easier to trigger AR.
- *Time points*: patients with AR are more likely to experience symptoms during 2 specific time points, morning and evening. Schenkel et al [62] assessed the severity of 4 nasal symptoms (sneezing, blockage, nasal runny nose, and nasal itch) at different times of the day, revealing that morning and evening symptoms were the most severe. This may be because of the circadian rhythm, pollen concentration, or personal behavior exacerbating the symptoms. In the evening, when the wind subsides, pollen settles closer to the ground and can be inhaled more easily. In addition, although humans rest at night in a horizontal position, nasal ventilation may be more difficult, leading to more severe symptoms. In the morning, low temperatures can cause congestion and swelling of the nasal mucosa because of the temperature difference between the environment and the body. This cluster had words such as “evening,” “get up early,” and “nose” as highly rated words.

This theoretical discussion regarding the potential pathway of risk factors that trigger AR can guide the development of detailed AR intervention measures. For example, patients with AR can pay attention to pollen concentration and temperature changes and adjust their outings and clothing accordingly based on the characteristics of the season; they can set the air conditioner to turn on or off based on their waking time to reduce the inhalation of cold air when waking up. Furthermore, they can adjust their sleeping position to reduce the frequency of nighttime symptoms.

## Discussion

### Principal Findings

This study aimed to identify the risk factors for AR based on social media comments. To do so, a data set of comments related to AR was collected, processed, and analyzed. The data set covered a consecutive period from May 2012 to May 2022. Overall, this analysis provided new insights into three main questions: (1) How many comments contained AR risk factor information? (2) How many categories can these risk factors be summarized into? (3) How do these risk factors trigger AR?

In assessing the identification of AR risk factors, we found that TopicS enhanced both precision and recall. TextCNN outperformed other models, achieving an accuracy of 0.9594 with a 150-dimension TopicS embedding. Analyzing 30,372 comments, our model pinpointed 5221 comments with risk factors. Categorizing the text items led to 28 distinct categories, with seasonal factors, regional variations, mites, weather changes, and unhealthy lifestyle habits emerging as common risks.

Furthermore, our research into AR risk factors revealed how risk factors trigger AR and uncovered the frequently reported, but underresearched, risk factors by affected individuals. Seasonal changes, especially during spring and autumn, increase exposure to pollen allergens, with varying climatic conditions affecting the development of allergies. Poor habits, such as smoking, irregular sleep, and frequent use of air conditioning, compromise immunity and heighten AR susceptibility. Dust mites, influenced by humidity, stand out as a primary allergen, with food items and indoor factors, such as animal dander, also triggering AR. Industrial pollutants and outdoor environmental factors amplify AR risk. Notably, AR symptoms intensify during mornings and evenings, which is likely influenced by circadian rhythms and environmental factors.

### Limitations and Future Work

This study has some limitations. Our study was based on the self-reported nature of social media data, and the lack of more detailed information from the study participants was a concern. Our statistics showed that seasonal factors, regional variations, mites, weather changes, and unhealthy lifestyle habits emerge as common risk factors, which is consistent with the findings of other studies based on surveys. Although social media may lack in-depth patient information, it provides an effective method of collecting breadth of data. Social media data can be gathered 24 hours a day and are an extremely efficient way to rapidly update new knowledge into the risk factor knowledge base. In the future, our framework can be expanded in 2 ways. First, the framework can track the development trends and changes in AR risk factors by leveraging real-time internet data sets. Second, the framework can be generalized and extended to detect patterns, trends, and risk factors for other chronic diseases such as type 2 diabetes.

### Conclusions

In this model improvement study, we proposed a topic-enhanced word-embedding model to improve the accuracy and recall of the text classification, namely to uncover less common or other types of risk factors based on social media data that have not been previously reported. The risk factors identified in this study can be a helpful reference for people with AR to reduce the development of the disease in their daily lives. This study establishes a knowledge base of potential risk factors for individuals who may not be aware of the factors that could trigger their symptoms. Patients can compare their lifestyle habits and medical history to identify their risk factors, which could help reduce the frequency of episodes and prevent the decline in their quality of life caused by blindly avoiding potential triggers. Our findings demonstrate the practicality and feasibility of using social media data for investigating disease knowledge. These findings may provide guidance for the development of management plans and interventions for AR.

## Appendix

Multimedia Appendix 1 Examples of social media text, topic dictionary examples, word-embedding dimension parameters with TextRNN, word-embedding dimension parameters with transformer, and social media category distribution and visualization.

Multimedia Appendix 2 Word cloud 1.

Multimedia Appendix 3 Word cloud 2.

Multimedia Appendix 4 Word cloud 3.

Multimedia Appendix 5 Word cloud 4.

Multimedia Appendix 6 Word cloud 5.

## Abbreviations

AR: allergic rhinitis
CBOW: Continuous Bag-of-Words Model
CNN: convolutional neural network
RNN: recurrent neural network

## Data Availability

The data sets generated and analyzed during this study are available from the corresponding author upon reasonable request.
