# Supplementary material for: Identifying the Risk Factors of Allergic Rhinitis Based on Zhihu Comment Data Using a Topic-Enhanced Word-Embedding Model: Mixed Method Study and Cluster Analysis
Source: J Med Internet Res. 2024 Feb 22;26:e48324. doi: 10.2196/48324 (PMC10921335; doi:10.2196/48324)
Supplement: Multimedia Appendix 1 [file jmir_v26i1e48324_app1.docx]

**Multimedia Appendix 1**

**Table S1.** Examples of social media text.

| No. | Text |
| --- | --- |
| 1 | I suffer from severe allergies during the spring and autumn seasons, and I sneeze when I feel temperature differences when getting up in the winter. |
| 2 | Dust mite allergy makes me feel like I’m dying every time it flares up. |
| 3 | When I’m standing or lying face down, my nose isn’t stuffy, but it gets the most congested when I’m lying flat. |
| 4 | I didn’t rest well because of the college entrance examination, and now whenever I stay up late at night, my nose gets stuffy. |
| 5 | My hometown is an industrial city with severe air pollution. When I was young, I had to go to the hospital every fall and winter. |
| 6 | I didn’t have allergic rhinitis before, but since giving birth in 2015, it has started to occur every late August every year. |
| 7 | This year feels slightly worse, and the pressure from work or study may be a significant contributing factor. |
| 8 | Because allergic rhinitis can be inherited and passed down to children, if parents have a history of allergic rhinitis, there is a high chance that their children will also develop allergic rhinitis. |

**Table S2.** Topic dictionary examples.

| Topic | Topic words (10 words given as examples) |
| --- | --- |
| Risk factors | Autumn, Winter, Pollen, Artemisia, City, Air pollution, Mites, Wuhan, Exercise, Diet |

Note: These English words or word combinations are one word in Chinese

**Table S3.** Word embedding dimension parameters with TextRNN

| Evaluation metrics | Model | 100 | 150 | 200 | 250 | 300 | 350 | 400 |
| --- | --- | --- | --- | --- | --- | --- | --- | --- |
| Accuracy  (%) | Skip-gram | 89.50 | 78.90 | 70.10 | 69.95 | 72.30 | 67.29 | 75.35 |
|  | TopicS^a^ | **93.90** | **91.35** | **91.15** | **91.40** | **87.55** | **77.20** | **80.05** |
| Precision  (%) | Skip-gram | 89.20 | 68.19 | 61.94 | 63.68 | 61.33 | 55.56 | 75.99 |
|  | TopicS | **91.20** | **76.90** | **77.40** | **81.50** | **75.20** | **76.40** | **65.70** |
| Recall  (%) | Skip-gram | 91.20 | 76.90 | 77.40 | 81.50 | 75.20 | 76.40 | 65.70 |
|  | TopicS | **90.14** | **71.37** | **65.63** | 70.14 | 66.40 | 63.23 | **65.92** |
| F1  (%) | Skip-gram | 90.14 | 71.37 | 65.63 | 70.14 | 66.40 | 63.23 | 65.92 |
|  | TopicS | **94.02** | **87.74** | **87.91** | **87.14** | **81.44** | **66.53** | **67.25** |

**Table S4.** Word embedding dimension parameters with Transformer

| Evaluation metrics | Model | 100 | 150 | 200 | 250 | 300 | 350 | 400 |
| --- | --- | --- | --- | --- | --- | --- | --- | --- |
| Accuracy  (%) | Skip-gram | 85.45 | 85.25 | 77.70 | 74.90 | 73.75 | 72.45 | 70.55 |
|  | TopicS^a^ | **89.00** | **90.70** | **75.95** | **76.10** | **75.95** | 72.15 | **75.35** |
| Precision  (%) | Skip-gram | 85.45 | 85.25 | 77.70 | 74.90 | 73.75 | 72.45 | 70.55 |
|  | TopicS | **89.00** | **90.70** | 75.95 | **76.10** | **75.95** | 72.15 | **75.35** |
| Recall  (%) | Skip-gram | 78.80 | 83.20 | 71.80 | 72.40 | 66.50 | 71.30 | 72.40 |
|  | TopicS | **89.90** | **90.60** | **72.30** | **75.30** | **75.60** | 70.90 | **73.00** |
| F1  (%) | Skip-gram | 81.13 | 84.63 | 75.77 | 74.15 | 70.54 | 71.44 | 70.89 |
|  | TopicS | **88.95** | **90.68** | **75.01** | **75.82** | **74.02** | **71.47** | **74.66** |

**Table S5.** Social media category distribution and visualization.

| No. | Class | Top 10 words (weight) | Word cloud | Number of text items |
| --- | --- | --- | --- | --- |
| 1 | Season | Summer (0.0313), inter (0.0311), season (0.021), season change (0.0141), spring (0.0139), autumn (0.0139), seasonal (0.0136), nose (0.0115), air condition (0.0108), month (0.0071) | 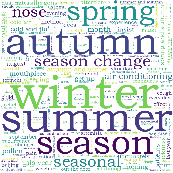 | 852 |
| 2 | Region | Beijing (0.0354), Shenzhen (0.0196), air (0.0105), Wuhan (0.0103), Guangdong (0.01001), city (0.0098), Shanghai (0.0093), dust mites (0.0092), nose (0.0091), university (0.0089) | 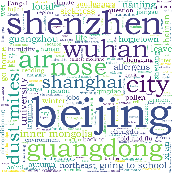 | 644 |
| 3 | Mites | Dust mites (0.111), mites (0.0447), dust (0.0202), allergy (0.0123), pollen (0.0085), allergens (0.0073), effect (0.0059), child (0.0047), nose (0.0046), cold air (0.0046) | 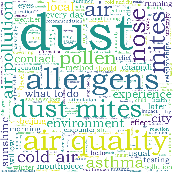 | 608 |
| 4 | Weather | Cold air (0.0709), weather (0.0233), temperature (0.0205), nose (0.0203), winter (0.0093), changes (0.0083), air (0.0082), alternate (0.0078), summer (0.0071), air condition (0.0071) | 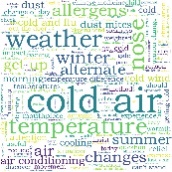 | 538 |
| 5 | Other disease | Cold and flu (0.0953), conjunctivitis (0.0194), urticaria (0.0165), nose (0.0102), asthma (0.0096), cough (0.0084), eczema (0.008), winter (0.0045), eyes (0.0045), physique (0.0044) | 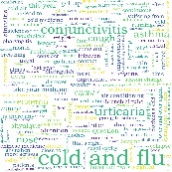 | 372 |
| 6 | Physique | Physique (0.0431), resistance (0.0309), immune system (0.022), immunity (0.0191), improve (0.01005), cold and flu (0.0094), question (0.0092), decline (0.00796), movement (0.0079), allergens (0.0065) | 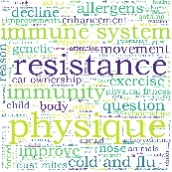 | 307 |
| 7 | Outdoors | Dust (0.0562), dust (0.039), air quality (0.0149), dust mites (0.0111), allergens (0.01009), nose (0.01006), mites (0.0099), air (0.0079), pollen (0.0076), allergens (0.0066) | 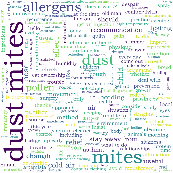 | 295 |
| 8 | Plant | Pollen (0.1004), artemisia (0.0258), dust mites (0.0159), ragweed (0.01), tarragon (0.01217), allergens (0.0101), plants (0.0088), artemisia (0.0075), allergens (0.0068), mites (0.0066) | 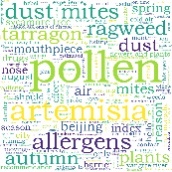 | 259 |
| 9 | Animal | Cat ownership (0.0427), animals (0.0144), asthma (0.0105), allergens (0.0103), allergens (0.0086), forced (0.0084), physique (0.0076), dust mites (0.007), experience (0.0073), cats (0.0065) | 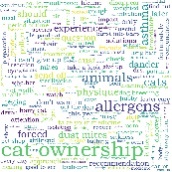 | 266 |
| 10 | Climate | Air (0.0319), local (0.0296), humidity (0.0239), climate (0.0203), dust mites (0.0197), wet (0.0189), nose (0.0167), raining (0.0138), weather (0.0132), environment (0.0128) | 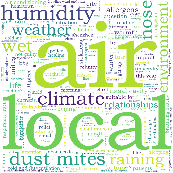 | 205 |
| 11 | Air conditioning | Air condition (0.1625), summer (0.0518), nose (0.0185), fans (0.01004), evening (0.0083), winter (0.0076), cold and flu (0.0067), cold air (0.0059), sleeping (0.0053), morning (0.0052) | 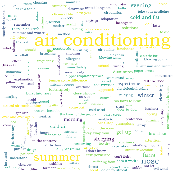 | 168 |
| 12 | Exercise | Running (0.08004), swimming (0.0406), insist (0.01701), movement (0.013980), nose (0.0117), winter (0.0098), finish step (0.0094), weighted (0.00699), evening (0.0064), exercise (0.0062) | 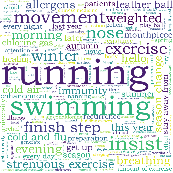 | 153 |
| 13 | Time | Morning (0.0332), evening (0.031), university (0.0214), get up early (0.0181), nose (0.0135), get up (0.0130), get up (0.0124), sleeping (0.0120), running (0.0069), air condition (0.0057) | 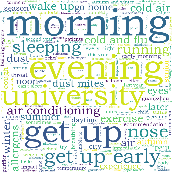 | 149 |
| 14 | Indoors | Go home (0.08), going to school (0.015), bed (0.0148), nose (0.0138), moving (0.0119), hair (0.0108), university (0.01), school (0.0097), Beijing (0.0085), cold and flu (0.0085) | 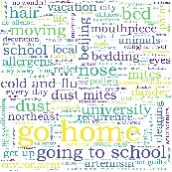 | 125 |
| 15 | Irritant gas | Scent (0.0313), smell (0.0238), vaccines (0.0153), formaldehyde (0.0125), pollen (0.0115), perfume (0.0114), dust (0.0104), stimulating (0.01), dust (0.0099), chlorine gas (0.0096) | 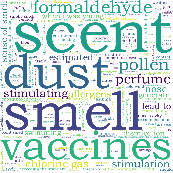 | 60 |
| 16 | Life habit | Stay up late (0.0498), smoking (0.0259), quit smoking (0.0226), nose (0.0225), living habits (0.0197), drinking (0.0175), weighted (0.015), resistance (0.0081), stay away from (0.0069), local (0.0067) | 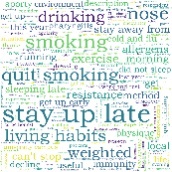 | 52 |
| 17 | Mental state | Spirituality (0.0558), nose (0.0149), cold air (0.0116), impact (0.01), too much (0.01), performance (0.01), jobs (0.0098), cold and flu (0.009), dust (0.0087), mental tension (0.0087) | 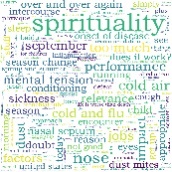 | 36 |
| 18 | FHH | Genetic (0.1181), physique (0.0454), factors (0.0145), the day after (0.0124), parents (0.0115), suffering from (0.011), cold and flu (0.01), child (0.01), family (0.0094), pregnancy preparation (0.0092) | 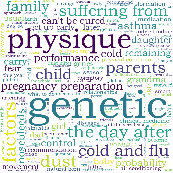 | 33 |
| 19 | Birth | Pregnancy (0.0556), child (0.0444), after birth (0.0346), pregnancy (0.0285), avoid (0.0122), seasonal (0.0122), winter (0.0118), postnatal (0.0105), spring (0.01045), this year (0.0096) | 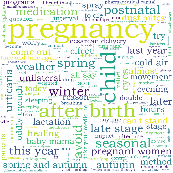 | 33 |
| 20 | Food | Milk (0.1044), eggs (0.0553), allergens (0.0376), seafood (0.0324), mold (0.0228), nose (0.0182), dust mites (0.0169), children (0.0149), mussel (0.0114), top (0.0114) | 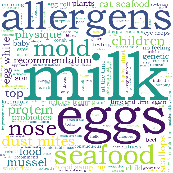 | 26 |
| 21 | Adherence | Stopping the medication (0.1241), automatic (0.0175), stop (0.0169), recurrence (0.0168), medication (0.0167), recover from (0.0151), weighted (0.0145), nose (0.012), want to (0.0099), take your medicine (0.0093) | 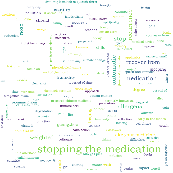 | 26 |
| 22 | Mold | Mold (0.1507), milk (0.0263), dust mites (0.0193), allergens (0.0177), child (0.0175), pollen (0.0143), air condition (0.0131), like (0.0131), avoid (0.0113), allergens (0.0112) | 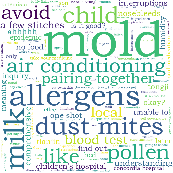 | 23 |
| 23 | Irritant diet | Mint (0.0329), evening (0.0272), extra spicy (0.0198), allergens (0.0194), isolated (0.0164), spicy and fishy (0.0142), morning (0.0139), toss (0.0135), stir-fry (0.0132), stop (0.0126) | 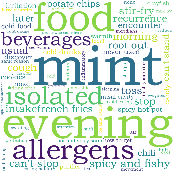 | 9 |
| 24 | Age | Age (0.1803), growth (0.0405), proliferation (0.0281), laboratory (0.0246), one nostril (0.0244), induced (0.0212), take your medicine (0.0207), weather (0.018), snore (0.0178), allergens (0.0163) | 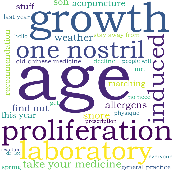 | 9 |
| 25 | HPI | Allergy history (0.0603), medical history (0.0595), past (0.0397), observation period (0.0389), defects (0.035), on time (0.0346), autumn (0.0283), going abroad (0.0258), acupuncture (0.0246), penicillin (0.0245) | 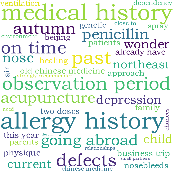 | 6 |
| 26 | Vaccine | Cockroach (0.0918), mites (0.053), subcutaneous injection (0.0272), allergens (0.0268), nose (0.02), every day (0.02), to die (0.0164), testing (0.0155), tropical (0.0155), come here (0.0147) | 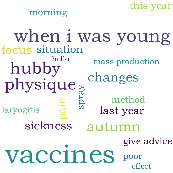 | 5 |
| 27 | Operation | Postoperative (0.1111), surgery (0.085), surgical procedure (0.0768), effect (0.0564), think (0.0492), recurrence (0.0433), ventilation (0.0429), nursing (0.0423), seven days (0.0415), exactly the same (0.0415) | 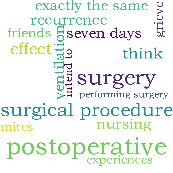 | 3 |
| 28 | Fungi | Fungi (0.2045), (of food) retain in stomach (0.1119), intestinal tract (0.1119), planting (0.095), originated from (0.095), infection (0.0926), performance (0.0783), tongue (0.0783), not really (0.0768) | 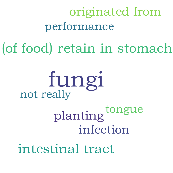 | 2 |
