# Supplementary figures and images for: Identifying the Risk Factors of Allergic Rhinitis Based on Zhihu Comment Data Using a Topic-Enhanced Word-Embedding Model: Mixed Method Study and Cluster Analysis
Source: J Med Internet Res. 2024 Feb 22;26:e48324. doi: 10.2196/48324 (PMC10921335; doi:10.2196/48324)

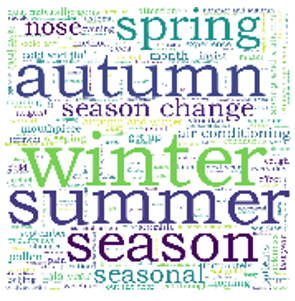

Supplement: Multimedia Appendix 2 [file jmir_v26i1e48324_app2.png]

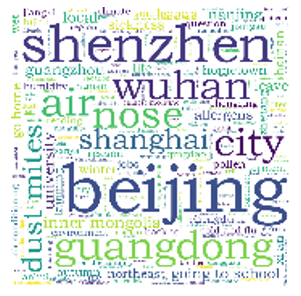

Supplement: Multimedia Appendix 3 [file jmir_v26i1e48324_app3.png]

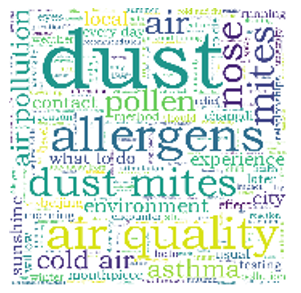

Supplement: Multimedia Appendix 4 [file jmir_v26i1e48324_app4.png]

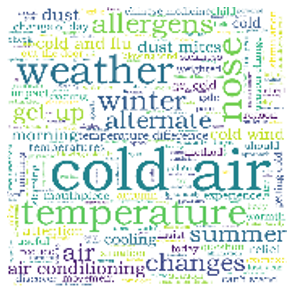

Supplement: Multimedia Appendix 5 [file jmir_v26i1e48324_app5.png]

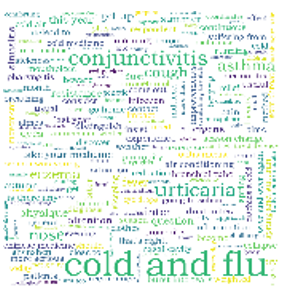

Supplement: Multimedia Appendix 6 [file jmir_v26i1e48324_app6.png]
